# Flagellar Motor Transformed: Biophysical Perspectives of the *Myxococcus xanthus* Gliding Mechanism

**DOI:** 10.3389/fmicb.2022.891694

**Published:** 2022-05-06

**Authors:** Jing Chen, Beiyan Nan

**Affiliations:** ^1^Department of Biological Sciences, Virginia Polytechnic Institute and State University, Blacksburg, VA, United States; ^2^Department of Biology, Texas A&M University, College Station, TX, United States

**Keywords:** myxobacteria, bacterial motility, bacterial gliding, proton channel, cell polarity, mechanosensing, force transmission

## Abstract

Many bacteria move on solid surfaces using gliding motility, without involvement of flagella or pili. Gliding of *Myxococcus xanthus* is powered by a proton channel homologous to the stators in the bacterial flagellar motor. Instead of being fixed in place and driving the rotation of a circular protein track like the flagellar basal body, the gliding machinery of *M. xanthus* travels the length of the cell along helical trajectories, while mechanically engaging with the substrate. Such movement entails a different molecular mechanism to generate propulsion on the cell. In this perspective, we will discuss the similarities and differences between the *M. xanthus* gliding machinery and bacterial flagellar motor, and use biophysical principles to generate hypotheses about the operating mechanism, efficiency, sensitivity to control, and mechanosensing of *M. xanthus* gliding.

## Introduction

Bacteria navigate their environments using diverse motility systems, yet many distinct mechanisms can find homology between each other, possibly as a result of divergent evolution. Such systems could use analogous energy sources and be subject to similar physical and chemical constraints. For example, rotary bacterial flagella, regardless of their localization (inside or outside of the cell wall) and energy sources (proton motive force or other ion gradient across the membrane), are all subject to comparable mechanochemical and hydrodynamic limits. In this perspective, we will focus on the gliding machinery of *Myxococcus xanthus*, which mediates linear cellular translocation on solid surfaces, yet uses a proton channel that is homologous to the energy-harvesting unit in rotary flagella (Nan et al., 2011; Sun et al., 2011).

**FIGURE 1:**
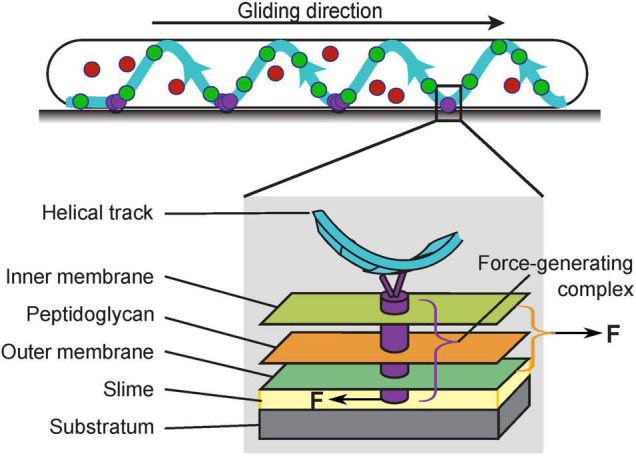
The gliding machinery of *Myxococcus xanthus*. The AglR/Q/S motors can be categorized into three subpopulations: those in the stationary force-generating complexes (purple), those undergoing directed motion along helical trajectories (green), and those that are diffusive (red). The fully assembled force-generating complexes span the entire cell envelop (gray box). To propel the cell forward, the force-generating complexes must exert opposite forces on the cell and substrate.

*Myxococcus xanthus* is a rod-shaped gram-negative bacterium that cannot swim due to the lack of flagella. Instead, it moves on surfaces using two motility systems, a social (S)-motility system that works more effectively in cells within groups, and an adventurous (A)-motility system that predominates in isolated cells. S-motility, also termed twitching motility, depends on extension and retraction of the Type IV pili (Wu and Kaiser, 1995; Chang et al., 2016), while A-motility, also termed gliding motility, is driven by a rather unique system. The entire gliding machinery consists of about 20 proteins and spans across the cytoplasm, inner membrane, peptidoglycan (PG) wall and outer membrane to interact with the substrate (Figure 1). The energy-harvesting unit in the inner membrane consists of AglR, AglQ, and AglS, which show significant homology with the MotA/B proteins in the flagellar stator of *Escherichia coli* (AglR is homologous to MotA, whereas AglQ and AglS are homologous to MotB; Nan et al., 2011; Sun et al., 2011). As a conserved proton-binding site on AglQ is essential for gliding, AglR/Q/S, similar to MotA/B, are predicted to form proton channels that convert the proton motive force into mechanical forces (Sun et al., 2011). For brevity, hereafter we will refer to the energy-harvesting proton channel as the “motor”, to be distinguished from the fully assembled gliding machinery. In contrast to the flagellar MotA/B stator, however, the AglR/Q/S motors are not fixed in the cell envelop, but rather travel the length of the cell along helical trajectories (Nan et al., 2011; Fu et al., 2018). When the motors travel to the sites where the cell contacts the substrate, they interact with the substrate through a group of Glt proteins that span the periplasm (Figure 1; Nan et al., 2010; Faure et al., 2016). In order to propel the cell forward, the motors must exert opposite mechanical forces on the substrate and the cell to cause relative motion between the two.

Single molecules of the motor and motor-associated proteins typically exhibit three subcellular dynamic patterns, stationary, directed motion, and diffusion. Current data suggest that the force-generating units are stationary, in which the motors assemble with other gliding proteins into fully functional gliding machineries (Nan et al., 2013; Faure et al., 2016; Nan, 2017). Most prominently, the stationary subpopulation aggregate in regularly spaced “focal adhesion” sites where the substrate interfaces with a helical intracellular track (Figure 1, purple circles; Nan et al., 2013). As the cell moves forward, these motor aggregations remain static with respect to the substrate; relative to the cell, they move toward the trailing pole at the speed of the cell (Mignot et al., 2007; Nan et al., 2010, 2013; Faure et al., 2016). Another subpopulation of motors travel along the helical track. The helical pattern of the motors (Figure 1, green circles) colocalizes with that of MreB, a bacterial cytoskeletal protein and homolog of eukaryotic actin. Disruption of MreB abolishes the helical motion of the motors and blocks gliding motility (Mauriello et al., 2010; Nan et al., 2011; Fu et al., 2018). However, it is yet unclear whether MreB directly provides the intracellular track for the motors, or rather, guides the helical motion of the motors indirectly. Although the helical motion of motors depends on the proton motive force and can reach ∼2 μm/s (Nan et al., 2013, 2015), the motors in directed motion could be in an intermediate state before the complete assembly of the force-generating units at the “focal adhesion” sites. Such incomplete machineries probably lack mechanical engagement with the substrate, and are thus not likely to provide propulsion for gliding (Nan et al., 2013; Faure et al., 2016; Nan, 2017). The third subpopulation of motor proteins are diffusive (Figure 1, red circles). As this subpopulation increases when either the proton motive force or MreB is disrupted^1^, it may be composed of incomplete motors, which are unable to harvest the proton motive force (Nan et al., 2013). Individual gliding proteins switch among the three states dynamically, out of which the switch into the stationary state likely reflects the assembly of the force-generating machineries and their engagement with the substrate. Notably, this assembly responds to substrate stiffness: the aggregation of stationary motors and motor-associated proteins intensifies on harder substrates and nearly all motors become stationary when the whole cell is embedded in the substrate (Nan et al., 2010, 2013). This phenomenon suggests that external mechanical cues can influence the gliding of *M. xanthus*, which is a hallmark of cellular mechanosensing.

Although the proteins constituting the *M. xanthus* gliding machinery are largely known, it remains unclear how the gliding machinery generates propulsion and drives linear motion of the cell. Based on prominent biophysical characteristics, such as the velocities of the motors and the intracellular helical track, a number of studies have proposed physical models for various aspects of gliding (Wolgemuth et al., 2002; Nan et al., 2011; Balagam et al., 2014; Tchoufag et al., 2019). As increasing amount of experimental data become available, we will review the similarities and differences between the gliding machinery in *M. xanthus* and the well-studied bacterial flagellar system in *E. coli*. Based on these comparisons, we will leverage biophysical principles to make hypotheses about the elusive mechanistic aspects of the *M. xanthus* gliding machinery, such as its assembly, force transmission, and sensitivity to control.

## Open Questions on the Mechanism of *M. Xanthus* Gliding

### How Does the Gliding Machinery Move Without Tearing the Cell Wall?

The envelop-spanning force-generating units at the “focal adhesion” sites are stationary relative to the substrate, while the cell wall moves forward at the cell velocity. Intuitively, the relative motion between the force-generating units and the cell wall would tear the wall and compromise its mechanical integrity. Yet, the cell wall is not broken in moving *M. xanthus* cells. How does the gliding machinery move without tearing the cell wall? This is a challenge that the statically positioned flagellar motor does not face.

**FIGURE 2:**
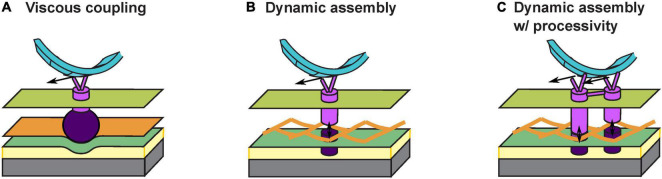
Models for interaction between the gliding machinery and substrate. **(A)** Viscous coupling between the gliding machinery and substrate. Large motor-associated proteins (dark purple) may deform the PG and the resulting bump exerts a viscous force on the substrate as the proton channel motor (bright purple) moves. **(B)** Dynamic assembly of the force-generating gliding machinery. An inner subcomplex (bright purple) that includes the motor and an outer subcomplex (dark purple) that binds with the substrate bind with each other dynamically across the PG mesh to allow force generation without tearing the PG. **(C)** Dynamic assembly of coupled gliding machineries confers processivity. The dynamic binding between the inner (bright purple) and outer (dark purple) subcomplexes is similar to **(B)**. Coordinated activities of coupled motors keep the motors on the helical track and/or at the “focal adhesion” sites for an extended period of time.

The answer to the above question depends on how the gliding machineries interact with the substrate. An early theory proposes a viscous coupling between the gliding machinery and the substrate, which entirely avoids penetrating the PG cell wall (Nan et al., 2011; Nan and Zusman, 2011; Tchoufag et al., 2019; Figure 2A). Particularly, some motor-associated proteins are of extraordinary sizes. For instance, AgmK, a transmembrane protein, contains 3,812 amino acids. Such proteins could form a bulky complex beneath the PG layer and deform the cell envelop locally. As the gliding machinery moves, the resulting bump at the surface of the cell would push against the extracellular slime, a putative layer of polysaccharides between the cell surface and the substrate (Figure 2A). The viscous drag from the slime thereby imposes thrust on the cell. This theory readily explains the correlation between the aggregation of motors at the “focal adhesion” sites and the stiffness of substrate: harder substrates presumably exert higher viscous drag on the gliding machineries and slow them down to create stronger aggregations (Nan et al., 2010, 2011, 2013). However, this viscous coupling cannot explain the cell’s resistance to sideway collisions (Balagam et al., 2014), unless the viscous drag between the cell and the substrate is highly anisotropic, with a much stronger drag perpendicular to the cell body. Currently, there is no evidence for such a strong anisotropy.

Recently, an updated theory proposes that the gliding machinery may consists of two subcomplexes inside and outside the PG wall, respectively (Faure et al., 2016; Figure 2B). The inner subcomplex contains the proton channel motor and the outer subcomplex directly binds the substrate. Transient association between the two subcomplexes across the mesh-like structure of PG (Hughes et al., 1975; Demchick and Koch, 1996; Vollmer et al., 2008) forms a fully assembled gliding machinery at the “focal adhesion” site. The fully assembly machinery is mechanically engaged with the substrate and generates propulsion (Figure 2B). Meanwhile, constant disassociation between the inner and outer subcomplexes allows the inner subcomplex to move relative to PG. Whereas this attractive hypothesis successfully circumvents the breach of PG, it also incurs a new problem: once the inner subcomplex disassociates from the outer subcomplex, without resistance from the substrate it may quickly escape from the “focal adhesion” site, causing a low duty ratio of the motor (i.e., the fraction of time a motor actually contributes to force generation). This also conflicts with the observation that most motors become stationary and stay engaged with the substrate under certain conditions, e.g., on very hard substrates (Nan et al., 2013). How do the inner and outer subcomplexes re-associate sufficiently fast across the PG so that the motors remain at the “focal adhesion” sites?

To address the above question, here we hypothesize that individual inner subcomplexes are coupled with each other: they bind with the outer subcomplexes alternately and hence help each other stay on the track (Figure 2C). Similar processive mechanisms are widely observed in well studied molecular motors, with the best example found in the hand-over-hand mechanism in dimeric kinesins (Yildiz et al., 2004; Gennerich and Vale, 2009). In *M. xanthus*, aggregation of motor proteins at the “focal adhesion” sites could reflect such coupling among multiple motors. Alternatively, the two MotB homologs, AglQ and AglS, may each constitute one of two coupled subunits, giving rise to a hand-over-hand mechanism within a single motor.

### How Do Motors Propel the Cell Body?

**FIGURE 3:**
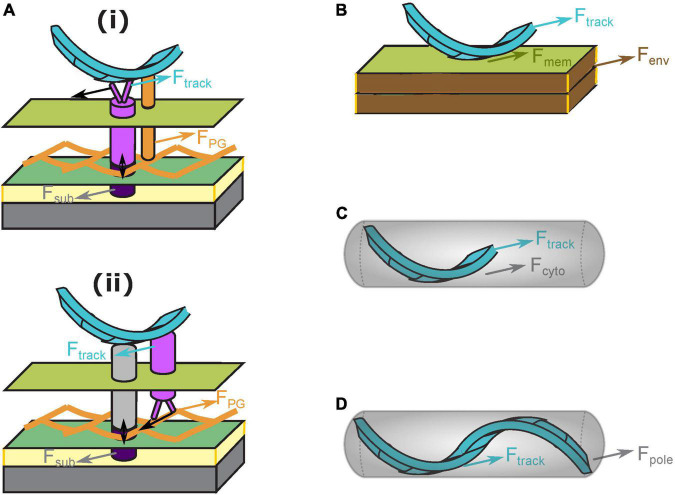
Hypotheses about transmission of motor force to the cell body. **(A)** Mechanical tethering between the intracellular track and cell envelop. The mechanical tethering can be mediated either through non-motor components of the gliding machinery **(i)** or directly through the motor components **(ii)**. In case **(ii)**, the “feet” of the motor are on the periplasmic side because the motor must travel with respect to the PG to stay static to the substrate. Bright purple: proton channel motor. Dark purple: outer subcomplex. Orange cylinder in case **(i)** mechanical tethering between the intracellular track and PG. Gray cylinder in case **(ii)** molecules connecting the intracellular track to the outer subcomplex (through dynamic binding/unbinding to avoid tearing the PG). Black arrows illustrate the moving direction of the motor. **(B)** Interaction between the intracellular track and cell membrane/envelop. **(C)** Viscous drag between the intracellular track and cytoplasm. **(D)** Direct push on the cell pole by the intracellular track.

As motors reside in the fluid membrane, they require a certain structure to transmit the force onto the cell body. Part of this structure must be localized inside the cytoplasm, such that the motors in the inner membrane can exert opposite forces on the cell and the substrate. The helical track along which the motors travel is a candidate for such a mechanical structure (Figure 1). But how exactly is the force transmitted? Generally speaking, the intracellular track may transmit force to the cell body in four ways (Figure 3): (i) through mechanical tethering with the cell envelop, (ii) through interaction with the membrane and cell envelop, (iii) through viscous drag against the cytoplasm, and (iv) by direct pushing against the cell pole. Note that the four mechanisms are not exclusive to each other, and may co-contribute to cell propulsion.

The first mechanism, tethering between the intracellular track and cell envelop, may effectively transmit the force from the former to the latter, thereby pushing the cell forward. Note that the intracellular track can tether to the cell envelop either indirectly through non-motor components of the gliding machinery, such as through the Rod PG synthesis complex associated with MreB (Garner, 2021; Rohs and Bernhardt, 2021; Figure 3A(i)) or directly through AglQ and AglS, if these proteins interact with PG mechanically (Figure 3A(ii)). In the former case, the motors push the intracellular track toward the leading pole, and the track in turn, pushes the PG forward. Whereas in the latter case, the intracellular track acts as a mechanical extension of the substrate and is pushed by the motors toward the trailing pole. These two mechanisms predict opposite directions of motion in the track relative to the cell, and can be tested by future experiments. In both cases, PG strands, if oriented helically, can guide the helical motion of the motors without MreB forming a continuous cytoplasmic track, as a recent report speculates (Faure et al., 2016); in the latter case, the PG even provides the direct track for the motors to move on.

The second mechanism, interaction between the intracellular track and cell membrane/envelop (Figure 3B), can effectively transmit the force from the intracellular track to the cell body, only if the interaction is sufficiently strong and the cell membrane exhibits glass-like properties in terms of its mechanical resistance against the motion of the track. The latter is possible if the membrane is crowded (Munguira et al., 2016). If the cell membrane behaves like fluid, as in *in vitro* membrane systems like the giant unilamellar vesicle (GUV), or the track interacts with the membrane too weakly, the mechanism would be insufficient for transmitting the motor force onto the cell. Verification of this hypothesis requires data on the physical properties and molecular components of the membranes in *M. xanthus*.

The third mechanism, viscous drag between the intracellular track and cytoplasm (Figure 3C), is unlikely to transmit significant force, because such a viscous drag is typically orders of magnitude lower than the force required to move a cell on a substrate surface (∼10^2^ pN (Wolgemuth, 2005; Sabass et al., 2017; Tchoufag et al., 2019). For example, a 10-μm microtubule traveling at ∼1 μm/s (observed speed of fluorescent MreB particles in *M. xanthus* (Fu et al., 2018) in the cytoplasm would encounter a sub-piconewton (pN) viscous drag force.

The fourth mechanism, the intracellular track directly pushing on the cell pole (Figure 3D), can only transmit the force effectively if the track is a continuous and relatively rigid structure, like the rotor ring in the bacterial flagellar motor. However, such a track may not exist, as MreB, the most likely constituent of the track, only forms patchy filaments (Fu et al., 2018), which probably lack the continuity and mechanical rigidity to generate a push on the cell pole.

### How Does the Gliding Machinery Switch Its Direction?

*M. xanthus* is known for periodic reversal of its direction of gliding, which is crucial for its “social” behaviors (Wu et al., 2007; Berleman and Kirby, 2009; Patra et al., 2016). Many previous models have investigated how intercellular coordination of gliding and reversals mediates formation of complex patterns and structures in the *M. xanthus* populations, e.g., rippling waves and fruiting bodies (Igoshin et al., 2001; Borner et al., 2002; Alber et al., 2004; Igoshin et al., 2004; Borner et al., 2006; Sliusarenko et al., 2007; Wu et al., 2009; Holmes et al., 2010; Harvey et al., 2012; Zhang et al., 2012a; Janulevicius et al., 2015; Patra et al., 2016). At the single-cell level, reversals are achieved by switching the cell’s polarity, which in turn, is determined by a group of polarity-setting molecules localized at the cell poles. Particularly, MglA is concentrated at the leading pole, and MglB, its antagonist, at the trailing pole (Leonardy et al., 2010; Zhang et al., 2010). MglA and MglB periodically switch between the two poles, causing cell polarity to switch (Leonardy et al., 2010; Zhang et al., 2010, 2012b; Keilberg and Sogaard-Andersen, 2014; Rashkov et al., 2014; Guzzo et al., 2018; Carreira et al., 2020). Moreover, the frequency of polarity switching is modulated by the Frz chemosensory pathway (Guzzo et al., 2018). This is reminiscent of the clockwise-counterclockwise switches in the bacterial flagellar motor, which is also controlled by the chemosensory pathway. We do not intend to discuss here the chemosensory pathway or polarity switching itself. Rather, we would like to raise the question of how the gliding machineries change the direction of force in response to the switched cell polarity.

**FIGURE 4:**
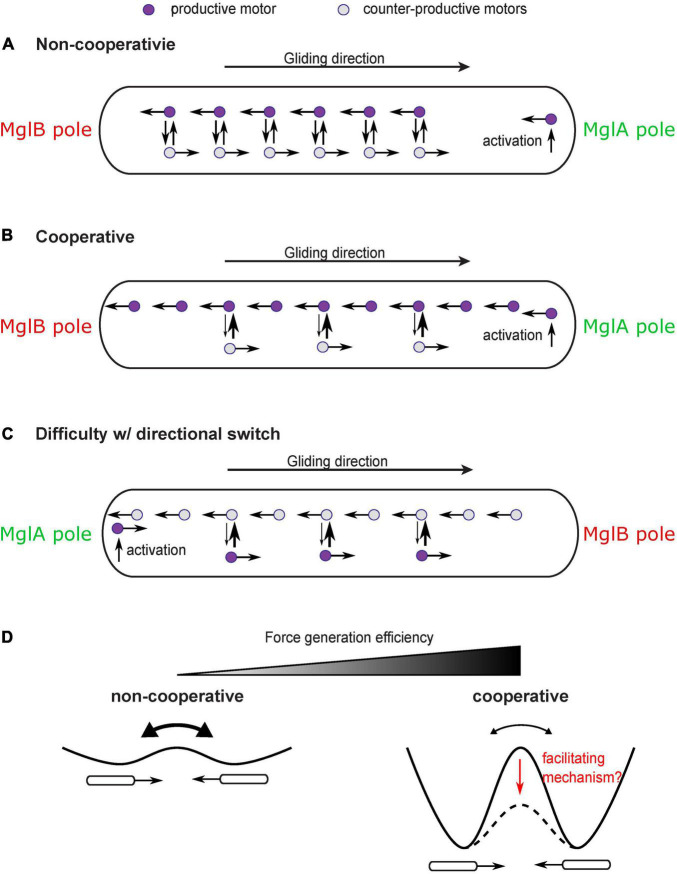
Cooperativity of gliding motors and issue with directional switch. **(A)** Constant reversal of gliding motors diminishes efficiency and stability of force generation. A large number of motors would travel in the counter-productive direction and produce forces in the counter-productive direction. **(B)** A cooperativity mechanism makes motors more likely to convert to the direction in which more motors are traveling, which enhances the bias toward productive motors and significantly increases the efficiency and stability of force generation. **(C)** The cooperativity mechanism may inhibit the reversal of the moving direction of the cell after the switch of cell polarity. The motors which are activated at the new MglA pole and travel in the new productive direction may be forced by the cooperativity mechanism to join the majority that remains traveling in the old productive direction. **(D)** Cooperativity among motors increases efficiency and stability of force generation at the price of inhibiting reversals. Reversals can be regarded as transitions between two symmetric states with equal energy. Cooperativity increases the energy barrier between the two states. A facilitating mechanism may be needed to reduce the energy barrier and promote state transition despite cooperativity.

This question may seem trivial at first sight, because MglA directly binds the gliding motor and likely mediates its activation (Nan et al., 2015; Treuner-Lange et al., 2015). One may envision that the gliding motor is activated at the MglA-concentrated pole and deactivated at the MglB-concentrated pole; traveling from the MglA pole to the MglB pole would generate a propulsion on the cell in the direction of the MglA pole. Hence, as the polar localization of MglA and MglB switches, the direction of propulsion also switches. This straightforward model, however, is challenged by the observation of constant reversals of single gliding motors as they travel along the intracellular track (Figure 4A; Nan et al., 2013, 2015). Due to the constant reversals, the gliding machinery cannot generate force consistently in one direction. Even though polarized activation and deactivation can bias the motion of gliding machineries in the productive direction (i.e., bound for the MglB pole), a large fraction of gliding machineries still generate counter-productive forces on the cell, with a 2∼20% estimated relative difference between motors in the two directions (the relative difference is roughly the ratio between the average time for motor reversal, ∼1 s (Nan et al., 2013, 2015) and the time for a motor to traverse the cell length without reversing, 5 s∼1 min (Sun et al., 2011; Nan et al., 2013, 2015). This makes the motility mechanism highly inefficient. With significant noise, this could also incur random switches of the cell’s direction of motion, which further reduces the efficiency of directional gliding.

In order to increase efficiency and stability of the gliding mechanism, a cooperativity mechanism is required to magnify the bias of motors in the productive direction (Figure 4B). For example, those gliding machineries traveling in the same direction as the cell may have a disadvantage in engaging with the substrate because they move at a higher speed relative to the substrate (relative speed to the cell + speed of cell). Therefore, the gliding machinery is more likely to stay in the productive direction. Cooperativity can also be boosted by intermolecular interactions among aggregated motors. In any case, the cooperativity can bring an asymmetry between motors traveling in opposite directions, which can magnify the directional bias induced by the established cell polarity and increase the fraction of productive gliding machineries over the counter-productive ones.

While significantly improving the efficiency and stability of force generation, such cooperativity has a down side: it may inhibit reversal of the cell’s moving direction even after cell polarity switches (Figure 4C). This is because the gliding machineries which are activated at the new MglA pole and travel in the new productive direction can be forced by the cooperativity mechanism to rejoin the majority that are still traveling in the old productive direction. The rate at which the new MglA pole generates productive motors is physically limited by the active motion and diffusion of motors. Therefore, it may not be strong enough to overcome the cooperativity mechanism and tip the balance in the new direction. Similar conflict between cooperativity and sensitivity of the directional switch was studied in the bacterial flagellar motor (Duke et al., 2001; Bai et al., 2010). Reversing the direction of rotation entails a switch of the C-ring—the rotor part of the flagellar motor (Chang et al., 2020). The switch requires cooperative conformational changes in all the rotor subunits that constitute the C-ring. Strong cooperativity among these subunits can suppress random switch of their conformation, promote structural homogeneity of the C-ring, and hence gauge the motor forces toward one direction. However, the cooperativity also imposes a challenge in switching the entire ring from one conformation to another in response to chemotactic signals (Duke et al., 2001; Bai et al., 2010). To circumvent this problem, Bai et al. (2010) proposed a mechanism: as a stator exerts force on a rotor subunit, it also promotes conformational changes in the latter and hence facilitate the switch of the rotor ring. It remains to be investigated if the force generated by the gliding machinery of *M. xanthus* plays a similar role in facilitating directional switch and overcoming the efficiency-boosting cooperativity (Figure 4D).

### How Does the Gliding Machinery Respond to Mechanical Load?

The observation that aggregations of gliding proteins at the “focal adhesion” sites intensify on harder substrates (Nan et al., 2010, 2013) implies that external mechanical cues can influence assembly and/or activity of the gliding machinery. This phenomenon is reminiscent of the observation that external load on the bacterial flagellar motor boosts the recruitment of stators into the motor complex (Lele et al., 2013; Tipping et al., 2013). The underlying molecular mechanism for the mechanosensing in the bacterial flagellar motor is known: the stators are dynamically recruited to and released from the motor and a “catch bond” mechanism retains the stators in the motor for longer time in response to a higher load (Nord et al., 2017; Wadhwa et al., 2019). Sharing homologous energy-harvesting proteins with the bacterial flagellar motor, the *M. xanthus* gliding machinery may exploit a similar force-sensitive mechanism that strengthens the engagement with the substrate upon higher load.

Interestingly, substrate stiffness also affects the reversal frequency of the *M. xanthus* cell (Zhou and Nan, 2017). It is possible that the gliding machinery further transduces the external mechanical cues into a signal that controls polarity switching. Currently, the most promising candidate that mediates the transduction from mechanical cues to regulatory signals for polarity switching is probably MglA, as it is not only a key molecule of the polarity-switching mechanism but also an essential component of the gliding machinery (Nan et al., 2015; Treuner-Lange et al., 2015). As the gliding machineries travel from the leading pole to the trailing pole, they carry the MglA along. The directed transport of MglA toward the trailing pole could affect the dynamics of polarity switching. Hence, the external mechanical cues could affect the reversal frequency through modulating the activity of the gliding machineries. Future investigations are needed to test this hypothesis.

## Conclusion

The *M. xanthus* gliding machinery exploits similar energy-harvesting units as the rotary flagellar motor, yet operates on a helical intracellular track. This is yet another testimony on the versatility of flagellar motors in biological systems. In this article, we brought up a few unresolved questions on the mechanism of *M. xanthus* gliding. As rotary motors are ubiquitous in nature and helical-tracking motors are also found in a number of bacteria (Nan et al., 2014), we predict that the answers to these questions will make wide impacts, much beyond the gliding motility in *M. xanthus*.

## Data Availability Statement

The original contributions presented in the study are included in the article/supplementary material, further inquiries can be directed to the corresponding authors.

## Author Contributions

JC and BN conceptualized and designed the work, curated and interpreted the relevant literature, and drafted the article. Both authors contributed to the article and approved the submitted version.

## Conflict of Interest

The authors declare that the research was conducted in the absence of any commercial or financial relationships that could be construed as a potential conflict of interest.

## Publisher’s Note

All claims expressed in this article are solely those of the authors and do not necessarily represent those of their affiliated organizations, or those of the publisher, the editors and the reviewers. Any product that may be evaluated in this article, or claim that may be made by its manufacturer, is not guaranteed or endorsed by the publisher.

## Footnotes

1 In Nan et al., 2013, the authors simplified the data analysis, in which particles are only categorized as stationary or in directed motion. The increase of the “slow-moving” particles actually corresponds to an increase in the diffusive population in a three-population analysis.
